# High diversity and unique composition of gut microbiomes in pygmy (*Kogia breviceps*) and dwarf (*K*. *sima*) sperm whales

**DOI:** 10.1038/s41598-017-07425-z

**Published:** 2017-08-03

**Authors:** Patrick M. Erwin, Ryan G. Rhodes, Kevin B. Kiser, Tiffany F. Keenan-Bateman, William A. McLellan, D. Ann Pabst

**Affiliations:** Department of Biology and Marine Biology, Center for Marine Science, University of North Carolina Wilmington, Wilmington, NC 28409 USA

## Abstract

Mammals host diverse bacterial and archaeal symbiont communities (i.e. microbiomes) that play important roles in digestive and immune system functioning, yet cetacean microbiomes remain largely unexplored, in part due to sample collection difficulties. Here, fecal samples from stranded pygmy (*Kogia breviceps*) and dwarf (*K*. *sima*) sperm whales were used to characterize the gut microbiomes of two closely-related species with similar diets. 16S rRNA gene sequencing revealed diverse microbial communities in kogiid whales dominated by Firmicutes and Bacteroidetes. Core symbiont taxa were affiliated with phylogenetic lineages capable of fermentative metabolism and sulfate respiration, indicating potential symbiont contributions to energy acquisition during prey digestion. The diversity and phylum-level composition of kogiid microbiomes differed from those previously reported in toothed whales, which exhibited low diversity communities dominated by Proteobacteria and Actinobacteria. Community structure analyses revealed distinct gut microbiomes in *K*. *breviceps* and *K*. *sima*, driven by differential relative abundances of shared taxa, and unique microbiomes in kogiid hosts compared to other toothed and baleen whales, driven by differences in symbiont membership. These results provide insight into the diversity, composition and structure of kogiid gut microbiomes and indicate that host identity plays an important role in structuring cetacean microbiomes, even at fine-scale taxonomic levels.

## Introduction

Microorganisms form symbiotic relationships with nearly all animal taxa, from basal invertebrate phyla (e.g. sponges)^1^ to humans^2^, and play important roles in the biology, ecology and evolution of animal life^3^. The structural and functional diversity of microbial communities (“microbiomes”) have been particularly well-studied in mammals and shown to be a key determinant of health and disease^4^. Indeed, host-microbe interactions in the mammalian gastrointestinal tract contribute significantly to host metabolism and pathogen defense. The gut microbiome provides essential nutrients (e.g. vitamins)^5^ and aids in the digestion of complex macromolecules to deliver energy sources otherwise unavailable to animal hosts^2^. Furthermore, these microbes are crucial to the development and regulation of the immune system^6^ and contribute to pathogen defense through colonization resistance, competing for colonization sites and secreting substances that inhibit pathogen growth^7^. The study of the complex bacterial and archaeal taxa that comprise mammalian gut microbiomes can thus provide insight into host health and homeostasis, and is particularly timely for species threatened by anthropogenic disturbances, such as marine mammals.

Marine mammals are emblematic and ecologically significant members of coastal and pelagic ecosystems^8^ that are represented in three mammalian orders (Carnivora, Cetartiodactyla and Sirenia), united by lifestyle rather than evolutionary history. The cumulative effects of direct (e.g. hunting) and indirect (e.g. habitat exploitation) anthropogenic impacts have led to worldwide declines in marine mammal populations, including extinction events^9^, and have resulted in over 20% of extant species being classified as threatened or endangered^10^. Mass mortality events from viral pathogens and secondary opportunistic infections^11, 12^ have prompted investigations of infectious diseases, their impacts of cetacean populations and their zoonotic implications^13^. In addition to the study of pathogenic bacteria and viruses, recent work has also focused on determining the composition and function of resident microbial communities inhabiting marine mammals. To date, most studies have focused on mammals in the orders Sirenia (e.g. dugongs^14, 15^, manatees^16^) and Carnivora (e.g. seals^17–19^, sea lions^20^), while cetaceans remain largely unexplored (whales, dolphins and porpoises, Order Cetartiodactyla)^21^, due in part to difficulties in *in situ* sample collection and the opportunistic nature of stranding events^22^. The emerging field of cetacean microbiology has largely focused on gut microbiome composition^20, 23, 24^, with recent metagenomic surveys characterizing the putative functionality of these symbiotic communities^25^. Preliminary trends in the structure of cetacean gut microbiomes include divergence between cetacean-associated and free-living microbial communities^20^, a high degree of host-specificity, and lower diversity microbiomes in toothed whale species compared to baleen whales^25^. Broader host taxon sampling is required to confirm these trends and apply this knowledge towards conservation and rehabilitation efforts for threatened and endangered cetacean species^26^.

In this study, we characterized the gut microbiomes of two closely-related species in the cetacean genus *Kogia*, *K*. *breviceps* (pygmy sperm whale) and *K*. *sima* (dwarf sperm whale), which exhibit similar gross morphology and ecological niches in open ocean habitats^27, 28^. Both species have a shark-like appearance, characterized by a blunt rostrum (or pointed snout), underslung jaw and cervical pigmentation pattern that resembles a gill slit^28^. Historically, the shared morphological characteristics between these species have confounded taxonomic analyses of the genus *Kogia*. Current species-level resolution of kogiid whales recognizes two species, based on morphology and molecular evidence^29–31^, although at-sea identification remains challenging. In general, *K*. *breviceps* exhibits greater body size than *K*. *sima*, with distinct differences in dorsal fin location, dorsal fin height and external “false gill slit” pigmentation between the two species^28, 31, 32^. It has been suggested that *K*. *breviceps* may dive more deeply and be found further offshore than *K*. *sima* ^32^, but these species display a high degree of dietary overlap that indicates very similar foraging ecologies^27^. Both species exhibit a worldwide distribution in tropical and temperate off-shore waters and predominantly feed on cephalopods during extended, deep dives. Observations at-sea of kogiid whales have been rare, due to their inconspicuous surface behavior and extended dive times, yet *K*. *breviceps* and *K*. *sima* are among the most commonly stranded cetaceans along the southeastern U.S.^33, 34^. Here, we examined fecal samples of stranded individuals of *K*. *breviceps* and *K*. *sima* to study kogiid gut microbiomes in a comparative context controlling for broad differences in diet and phylogenetic history. Our objectives were to provide the first characterization of kogiid gut microbiomes and to determine their host-specificity, by comparing microbiome diversity and structure between kogiid species and across other toothed (odontocete) and baleen (mysticete) whale hosts. We hypothesized that kogiid gut microbiomes would exhibit: (1) similar richness and diversity compared to other toothed whale species, (2) lower richness and diversity compared to baleen whale species, and (3) distinct community structure compared to other toothed and baleen whale species.

## Results

### Composition of cetacean gut microbiomes

**Figure 1:**
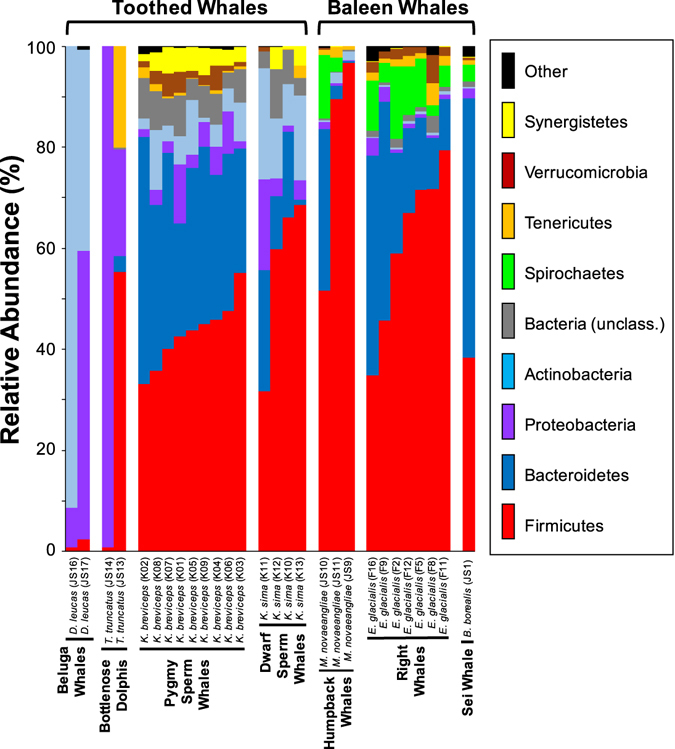
Phylum-level composition of gut microbiomes in *Kogia breviceps* and *K*. *sima* compared with previous data from other toothed and baleen whales^25^. Relative abundances of common microbial phyla are shown in color, with rare phyla (other) in black (Fusobacteria, Euryarchaeota, Lentisphaerae, Cyanobacteria, Planctomycetes, Fibrobacteres, Deferribacteres, TM7, TM6, Acidobacteria, Elusimicrobia, Armatimonadetes, Thermi, and Thermotogae).

A total of 1,720 symbiont OTUs were recovered from *K*. *breviceps* (*n* = 1,368) and *K*. *sima* (*n* = 890), representing 11 bacterial phyla and one archaeal lineage (Fig. 1, Table S1). The gut microbiomes of *K*. *breviceps* and *K*. *sima* were dominated by the bacterial phyla Firmicutes and Bacteroidetes, together accounting for >68% of the total gut microbial communities in each kogiid host. Actinobacteria, Proteobacteria, Synergistetes and Verrucomicrobia were also common members of kogiid gut microbiomes (Fig. 1), with six additional rare phyla (<1% relative abundance) detected in both *K*. *breviceps* and *K*. *sima* (Table S1). Notably, some differentiation of gut microbiomes in *K*. *breviceps* and *K*. *sima* was observed at the phylum level. In total, five phyla exhibited differential abundances between hosts, including significantly (*P* < 0.05) higher relative abundances of Bacteroidetes, Verrucomicrobia and Lentisphaerae in *K*. *breviceps*, and Actinobacteria and Cyanobacteria in *K*. *sima* (Table S1).

Comparative analysis with other toothed and baleen whale species revealed that the dominance of the bacterial phyla Firmicutes and Bacteroidetes in kogiid microbiomes was more characteristic of baleen whales than those of other toothed whales (Fig. 1). The vast majority (84 to 91%) of the gut microbiomes in baleen whale hosts (humpback whales, right whales, and sei whales) were comprised of Firmicutes and Bacteroidetes, whereas non-kogiid toothed whale hosts were dominated by Proteobacteria (60% in bottlenose dolphins) and Actinobacteria (66% in beluga whales, Fig. 1). Despite these shared dominant phyla, several bacterial phyla distinguished kogiid microbiomes from those in baleen whales: Actinobacteria and Synergistetes were common in kogiid hosts (6–15 and 2–4%, respectively) while rare in baleen hosts (<2 and <0.2%, respectively), while Spirochaetes were common members of baleen whale microbiomes (3–8%) and extremely rare in kogiid hosts (<0.03%).

### Diversity of cetacean gut microbiomes

**Figure 2:**
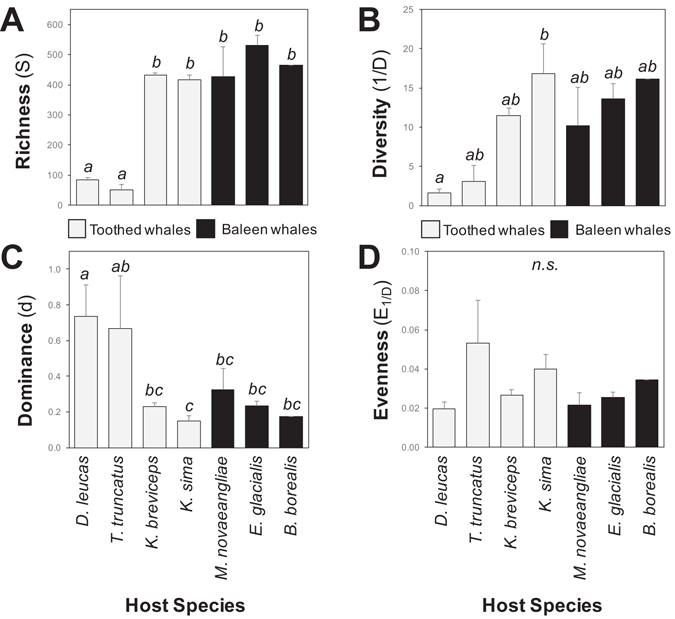
Diversity metrics of kogiid gut microbiomes compared with previous data from other toothed (*gray bars*) and baleen (*black bars*) whales^25^. Different letters above bars denote significantly different means among host species (*P* < 0.05). No significant differences in evenness occurred between host species (*n*.*s*. = not significant). Error bars represent ±1 standard deviation.

Gut microbiomes in kogiid whales exhibited high OTU-level diversity, averaging 432 (±7 SE) and 416 (±18 SE) symbiont OTUs per host in *K*. *breviceps* and *K*. *sima*, respectively. No significant differences in richness (*S*), diversity (*1/D*), evenness (*E*_*1/D*_) and dominance (*d*) were observed in gut microbial communities between kogiid hosts (*post hoc* Tukey’s HSD tests, Fig. 2), indicating similar richness and evenness of gut microbiomes in these closely related species. Broader comparisons of diversity including previously characterized cetacean gut microbiomes revealed that kogiid whales hosted microbial communities with significantly (*P* < 0.05) higher diversity than those occurring in other toothed whales (bottlenose dolphins = 52 ± 18 OTUs, beluga whales = 84 ± 8, *post hoc* Tukey’s HSD tests, Fig. 2). Similarly, a single OTU dominated the microbial communities of bottlenose dolphins (67%) and beluga whales (74%), while the most abundant OTU accounted for less than a quarter of the microbiome in *K*. *breviceps* (23%) and *K*. *sima* (15%). In contrast, kogiid gut microbiomes did not differ significantly in richness, diversity and dominance compared to the gut microbial communities inhabiting three baleen whale species (*post hoc* Tukey’s HSD tests, Fig. 2).

### Community structure of cetacean gut microbiomes

**Figure 3:**
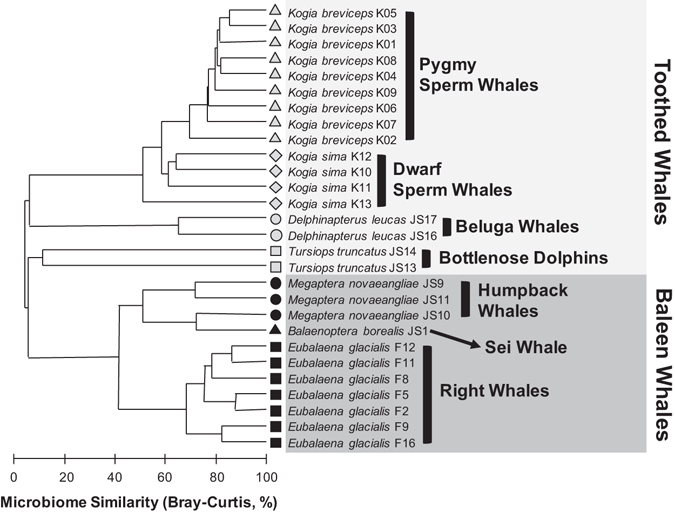
Similarity of gut microbiomes in *Kogia breviceps* and *K*. *sima* compared with previous data from other toothed and baleen whales^25^ based on relative abundance OTU data. Shaded boxes delineate toothed whale species (*light gray*) and baleen whale species (*dark gray*).

Despite similarities in community diversity, the gut microbiomes in *K*. *breviceps* and *K*. *sima* exhibited significant differences in community structure according to both OTU-based and phylogenetic beta-diversity metrics (PERMANOVA, *P* < 0.01, Table 1), indicating that distinct microbial communities inhabit each host species. These differences were visible in cluster plots based on microbiome similarity, which grouped kogiid individuals by host species (Fig. 3). Importantly, these structural differences were driven by differences in the relative abundance of shared symbiont taxa, as analyses based on symbiont membership revealed no significant differences (PERMANOVA, *P* > 0.11) in microbiome community structure between kogiid hosts for either beta-diversity metric (Table 1, Fig. S1) and no host-specific clustering of microbiomes in cluster plots (Fig. S1). Indeed, the 538 OTUs shared between *K*. *breviceps* and *K*. *sima* accounted for >99% of the recovered sequences, while the unique OTUs in each hosts’ microbiome (*n* = 830 in *K*. *breviceps*, *n* = 352 in *K*. *sima*) were extremely rare (in total, representing <1% of all sequences). Significant differences in dispersion were detected between the microbiomes of *K*. *breviceps* and *K*. *sima* (PERMDISP, *P* < 0.01, Table S2), indicating greater intra-specific variability in microbial communities among *K*. *sima* individuals. Again, these differences were driven by variability in the relative abundance of shared symbionts, as analyses based on symbiont membership revealed no significant differences in dispersion (PERMDISP, *P* > 0.05, Table S2). No significant differences in the structure of kogiid gut microbiomes were detected based on sex (ANOSIM, *P* > 0.18) or carcass condition (*P* > 0.36, Table S3).

**Table 1:** Pairwise statistical comparisons of microbial community structure (PERMANOVA) across cetacean hosts, based on OTU-dependent (Bray Curtis) and OTU-independent (UniFrac) metrics of relative abundance (Rel. Abund., Weighted) and presence-absence (Presence-Abs., Unweighted) data.

Pairwise Comparison	Bray-Curtis Similarity				UniFrac Distance			
	Rel. Abund.		Presence-Abs.		Weighted		Unweighted	
	*t*	*P*	*t*	*P*	*t*	*P*	*t*	*P*
*K*. *breviceps* - *K*. *sima*	2.405	0.001*	1.350	0.119	2.906	0.001*	1.187	0.145
*K*. *breviceps* - *D*. *leucas*	6.531	0.001*	15.29	0.001*	5.702	0.001*	2.228	0.003*
*K*. *breviceps* - *E*. *glacialis*	10.405	0.001*	46.88	0.001*	5.006	0.001*	3.085	0.001*
*K*. *breviceps* - *M*. *novaeangliae*	6.895	0.001*	25.44	0.001*	3.681	0.002*	2.304	0.002*
*K*. *breviceps* - *T*. *truncatus*	4.045	0.001*	8.223	0.001*	3.287	0.001*	2.143	0.003*
*K*. *sima* - *D*. *leucas*	3.509	0.005*	9.296	0.001*	2.844	0.010*	1.856	0.033
*K*. *sima* - *M*. *novaeangliae*	3.854	0.001*	15.827	0.001*	1.927	0.025	1.900	0.025
*K*. *sima* - *E*. *glacialis*	6.071	0.001*	30.62	0.001*	3.014	0.001*	2.447	0.001*
*K*. *sima* - *T*. *truncatus*	2.270	0.022	4.927	0.002*	1.704	0.065	1.770	0.034
*E*. *glacialis* - *D*. *leucas*	5.831	0.001*	11.558	0.001*	3.877	0.001*	2.061	0.005*
*E*. *glacialis* - *M*. *novaeangliae*	3.440	0.001*	7.181	0.001*	1.609	0.079	1.625	0.029
*E*. *glacialis* - *T*. *truncatus*	3.495	0.004*	6.253	0.001*	2.633	0.002*	1.968	0.007*
*M*. *novaeangliae* - *D*. *leucas*	3.788	0.004*	6.231	0.002*	2.337	0.032	1.553	0.098
*M*. *novaeangliae* - *T*. *truncatus*	1.964	0.070	3.122	0.014*	1.530	0.136	1.445	0.174
*T*. *truncatus* - *D*. *leucas*	1.770	0.141	2.321	0.075	1.121	0.379	1.108	0.386

Asterisks (*) indicate significant differences following B-Y corrections.

The high degree of host-specificity exhibited by kogiid gut microbiomes was also observed across more distantly related host species, including other toothed whales and baleen whales, as cluster plots based on microbial community similarity grouped all individuals by host species (Fig. 3). Host species accounted for the majority (69%) of variation observed between samples and significant differences between hosts were observed for most pairwise comparisons (Table 1), with exceptions generally involving pairwise comparisons with low statistical power (i.e. hosts with low replication, *T*. *truncatus* and *D*. *leucas*). Differences between the gut microbiomes of these more distantly related cetacean hosts were driven by symbiont membership, rather than relative abundance of shared taxa alone, as analyses of presence-absence data revealed a higher proportion of variation (83%) explained by host species and more distinct clustering of host species in cluster plots (Fig. S1).

### Dominant symbiont OTUs in kogiid gut microbiomes

Core microbiomes in kogiid whales were identified as symbiont OTUs detected in all samples of each host species, thereby excluding potentially transient members of the recovered communities. Core microbiomes were comprised of 147 and 155 OTUs in *K*. *breviceps* and *K*. *sima*, respectively, and represented a small fraction of overall symbiont diversity (10.7% in *K*. *breviceps*, 17.4% in *K*. *sima*). However, core OTUs also represented dominant symbiont taxa and accounted for the vast majority of overall symbiont communities (96.2 to 97.4%). A high degree of overlap in symbiont membership was observed when comparing the core communities in *K*. *breviceps* and *K*. *sima*. The shared component of core microbiomes in these two kogiid hosts was comprised of 115 OTUs that accounted for most of the sequence reads recovered from *K*. *breviceps* (95.6%) and *K*. *sima* (94.0%, Table S4).

The 25 most common OTUs in kogiid microbiomes were examined in further detail to compare their relative abundances between kogiid hosts and presence in the microbiomes of other toothed and baleen whales (Table 2). All 25 OTUs represented core members of both kogiid species microbiomes, yet exhibited different relative abundances in each host. Together, these 25 OTUs contributed to 66.3% of the variation observed between the microbiomes of *K*. *breviceps* and *K*. *sima*, with 14 OTUs (56%) exhibiting significantly different abundances between kogiid hosts (Table 2). Eighteen (72.0%) of these OTUs were unique to kogiid microbiomes (i.e., not detected in the other toothed and baleen hosts included herein) and 15 OTUs (60%) exhibited greater than 3% sequence divergence from any previously characterized sequence (GenBank database), likely representing novel bacterial species. Combined with the unresolved taxonomic status of some dominant OTUs, these results indicate that several novel taxa comprise kogiid microbiomes and are most closely related to symbionts identified in gut communities of other mammalian hosts (Table 2). For example, the most abundant OTU in both kogiid hosts (OTU1) was unique to kogiid microbiomes, unclassified below the phylum level (Bacteroidetes) and exhibited over 9% sequence divergence from the most closely related bacterium, a symbiont characterized from elephant (*Elephas maximus*) feces (Table 2).

**Table 2:** Common OTUs in the gut microbiomes of *Kogia breviceps* and *K*. *sima*, showing phylum- and lowest-level taxonomy, relative abundances, and percentage contributions to dissimilarity between hosts (SIMPER analysis).

OTU	Phylum (lowest taxonomy)	*K*. *breviceps*	*K*. *sima*	% Contrib.	Unique	BLAST Match Source (Identity, Acc. No.)
*000001	Bacteroidetes (unclassified)	22.98 ± 2.12	9.72 ± 3.66	10.87	Yes	Elephant Feces (90.6, EU471682)
*000007	Firmicutes (o_Clostridiales)	3.33 ± 1.29	7.09 ± 1.28	4.14	Yes	Dolphin Rectum (96.0, JQ204280)
*000010	Firmicutes (f_Mogibacteriaceae)	5.07 ± 0.46	1.64 ± 0.95	1.97	Yes	Sea Lion Rectum (94.0, JQ207359)
*000018	Synergistetes (f_Synergistaceae)	3.78 ± 0.43	0.14 ± 0.08	2.94	Yes	Zebra Feces (92.4, EU470284)
*000021	Bacteroidetes (o_Bacteroidales)	3.22 ± 0.62	0.10 ± 0.04	2.52	Yes	Bovine Colon (90.5, JX096352)
*000008	Firmicutes (g_*Oscillospira*)	2.55 ± 0.45	0.97 ± 0.34	1.34	No	Equine Manure (100, AY212772)
*000026	Proteobacteria (*Campylobacter fetus*)	2.62 ± 1.41	0.04 ± 0.00	2.08	Yes	Human Feces (99.2, CP015575)
*000030	Verrucomicrobia (f_RFP12)	2.26 ± 0.53	0.11 ± 0.07	1.73	Yes	Sediment (92.6, GU453511)
*000020	Actinobacteria (f_Coriobacteriaceae)	0.25 ± 0.03	4.11 ± 1.85	3.1	Yes	Bovine Rumen (91.7, KT172105)
*000022	Actinobacteria (g_*Adlercreutzia*)	0.40 ± 0.05	3.63 ± 1.08	2.59	Yes	Swine Feces (93.3, KP102484)
*000039	Firmicutes (f_Ruminococcaceae)	1.57 ± 0.52	0.24 ± 0.06	1.07	Yes	Chicken Feces (96.0, JQ248085)
*000038	Firmicutes (g_*Butyrivibrio*)	1.52 ± 0.20	0.29 ± 0.04	0.99	Yes	Sea Lion Rectum (96.0, JQ208575)
*000047	Bacteria (unclassified)	1.40 ± 0.52	0.05 ± 0.01	1.1	Yes	Anaerobic Digester (90.1, KF631052)
*000058	Firmicutes (o_Clostridiales)	1.17 ± 0.81	0.05 ± 0.01	0.92	Yes	Swine Feces (96.5, KP107340)
000002	Firmicutes (f_Peptostreptococcaceae)	10.20 ± 2.29	6.67 ± 3.35	6.17	No	Dolphin Rectum (98.8, JQ202598)
000005	Firmicutes (*Clostridium perfringens*)	1.65 ± 0.86	8.63 ± 5.37	6.79	No	Human Feces (100, KX674026)
000004	Actinobacteria (*Mycobacterium arupense*)	3.83 ± 1.27	1.32 ± 0.56	2.7	No	Porpoise Feces (100, JN792395)
000013	Bacteria (unclassified)	1.32 ± 0.43	3.81 ± 1.86	2.6	Yes	Dolphin Rectum (98.8, JQ203364)
000025	Bacteroidetes (unclassified)	1.92 ± 0.78	1.57 ± 1.42	1.89	Yes	Bovine Rumen (92.1, AB616513)
000016	Firmicutes (g_*Oscillospira*)	1.45 ± 0.21	2.38 ± 1.47	1.51	Yes	Human Feces (100, HQ808319)
000034	Bacteroidetes (unclassified)	1.52 ± 0.40	0.39 ± 0.21	1.02	Yes	Bovine Rumen (90.2, GQ327094)
000033	Proteobacteria (g_*Citrobacter*)	0.10 ± 0.04	3.29 ± 3.23	2.63	No	Human Feces (100, CP016762)
000037	Firmicutes (f_Mogibacteriaceae)	0.78 ± 0.06	1.63 ± 0.82	0.92	Yes	Sea Lion Rectum (96.4, JQ207359)
000029	Firmicutes (*Clostridium perfringens*)	0.08 ± 0.03	2.60 ± 2.10	2.06	No	Dolphin Rectum (100, JQ202064)
000049	Firmicutes (*Faecalibacterium prausnitzii*)	0.94 ± 0.24	0.61 ± 0.42	0.68	No	Seal Colon (99.2, GQ867580)

Unique OTUs were those not detected in other whale species (comparative analysis herein). BLAST matches show the source and identity (%) of the closest known relative for each OTU. Asterisks (*) denote differentially abundant OTUs between kogiid hosts.

The few symbiont OTUs shared between gut communities of kogiid and other cetacean hosts were generally in very low abundance in one or both hosts, with notable exceptions. OTU2 (Firmicutes, Peptostreptococcaceae) was a dominant member of the microbiomes in both kogiid hosts (10.2% in *K*. *breviceps*, 6.7% in *K*. *sima*) as well as bottlenose dolphins (18.5%). *Mycobacterium arupense* (Actinobacteria, OTU4) and *Oscillospira* sp. (Firmicutes, OTU8) were common in *K*. *breviceps* (3.8 and 2.6%, respectively) and *K*. *sima* (1.3 and 1.0%), with the former symbiont a dominant member of beluga whale microbiomes (65.3%) and the latter symbiont a dominant member of humpback whale microbiomes (14.5%).

## Discussion

Characterization of gut microbiomes in pygmy (*K*. *breviceps*) and dwarf (*K*. *sima*) sperm whales revealed diverse and host-specific microbial communities inhabiting kogiid whales. Gut microbiomes in *K*. *breviceps* and *K*. *sima* exhibited similar levels of richness and evenness, along with high overlap in symbiont membership, yet were differentiated by the relative abundance of shared symbiont taxa. As a result, distinct microbial community structure was detected between kogiid species, as well as, among kogiid microbial communities and previously characterized gut microbiomes of other toothed and baleen whales. Notably, these latter differences were driven by differences in community membership and several dominant kogiid-associated OTUs exhibited high levels of differentiation from any described bacteria, indicating the presence of unique symbiont taxa in these hosts. Kogiid gut microbiomes exhibited unique structure and composition and a high-degree of host-specificity, thus highlighting the importance of host identity in structuring cetacean microbiomes.

The diversity of gut microbial communities in kogiid whales was unexpectedly high, as previous investigations of toothed whale gut microbiomes revealed low levels of phylum- and OTU-level diversity. Symbiont OTU richness in *K*. *breviceps* (432 ± 7) and *K*. *sima* (416 ± 18) was nearly five times higher than the gut microbiomes of bottlenose dolphins (*T*. *truncatus* 52 ± 8) and beluga whales (*D*. *leucas* 84 ± 18) included in comparative analyses herein. In contrast to the kogiid colonic content sampled from stranded wild individuals, fecal samples from bottlenose dolphins and beluga whales were collected following defecation by captive individuals^25^. However, similarly low diversity communities have been reported in other toothed whale species, including rectal swabs of wild (36 ± 2) and captive (41 ± 8) bottlenose dolphins^20^, striped dolphins (*Stenella coeruleoalba*, 75 OTUs)^23^ and colonic contents of the Yangtze finless porpoise (*Neophocaena phocaenoides asiaeorientalis*, 30 OTUs)^24^, indicating that different sampling methods and host status (wild vs. captive) alone do not account for the observed differences in microbiome richness between kogiids and other toothed whales. As such, kogiid whales appear to represent a more fertile microbial habitat compared to other toothed whale species, possibly related to the unique colonic sac that enlarges the large intestine of kogiid whales (e.g. 50 L of feces were extracted from a 295 cm individual *K*. *breviceps*)^35^. The presence of this colonic feature, and its potential to sequester feces for extended periods of time, are also hypothesized to contribute to the detectability of the neurotoxin domoic acid in kogiids^36^. Otherwise, kogiid gastrointestinal tracts exhibit similar morphology to those of most other odontocetes (including *Physeter macrocephalus*) and mysticetes, with a multi-chambered stomach consisting of single fore-, main and pyloric chambers^37^. Interestingly, the low diversity of gut microbiomes in non-kogiid toothed whales contrasts with other body sites of these animals, where high diversity microbial communities have been reported. For example, the oral microbiome of bottlenose dolphins displayed twice the phylum-level and four times the OTU-level diversity compared to their gut microbiomes^20^. Whether the observed differences in microbiome diversity between kogiid and other toothed whales translate into differences in community functionality and stability (e.g. via functional redundancy) are important questions for understanding host-symbiont interactions and will require additional metagenomic and cultivation-based studies of whale microbiomes.

Kogiid whale gut microbiomes were dominated by symbiont taxa affiliated with the phyla Firmicutes and Bacteroidetes, two common lineages in mammalian gut communities. Members of the phylum Firmicutes were rare in some non-kogiid toothed whale hosts, for example, representing <2% of gut communities in beluga whales. In bottlenose dolphins, high variability was observed in the relative abundance of Firmicutes symbionts (<1 to 55%), though recent work indicates that Firmicutes are indeed dominant members of the bottlenose dolphin gut microbiome^20, 38^. In contrast, Bacteroidetes were greatly reduced (<2%) in gut microbiomes of both non-kogiid toothed whales investigated herein, consistent with previous studies^20, 38^, while comprising a large fraction of symbiont communities in *K*. *breviceps* (31%) and *K*. *sima* (13%). Bacteroidetes are common members of herbivorous^14–16^ and carnivorous^18, 39, 40^ marine mammal microbiomes and are well known for their ability to degrade high molecular weight organic compounds, including complex polysaccharides^41^. Accordingly, these symbionts are thought to play a key role in the digestive health of mammals and their absence in many toothed whale species may reflect alternative nutrient acquisition strategies. Importantly, Bacteroidetes have been reported as common (even dominant) in other body sites of toothed whale hosts, including the oral, upper gastric and respiratory microbiomes of bottlenose dolphins^20^, thus their absence in the lower gut may indicate environmental selection against these taxa in this particular microhabitat of these hosts.

The structural determinants of gut microbiome composition in marine mammals include age, diet and phylogenetic position^17, 20, 25^, similar to studies of terrestrial counterparts^42, 43^. In this study, the similar foraging niches and dietary overlap between *K*. *breviceps* and *K*. *sima* ^27^ allowed for the investigation of cetacean microbiomes in a comparative context controlling for broad differences in diet and isolating host-specific factors. Our results suggest that host identity plays an important role in structuring cetacean microbiomes, even at fine-scale taxonomic levels, as distinct microbiome structure was observed between closely-related kogiid hosts. In contrast, host sex and carcass condition showed no significant effects on microbiome structure in kogiid whales. Evidence to date indicates that sex is not an important structuring factor for marine mammal microbiomes, as past studies have similarly reported no significant effect of sex on gut microbiomes of bottlenose dolphins^20^, leopard seals (*Hydrurga leptonyx*)^17^, manatees (*Trichechus manatus*)^16^ and dugongs (*Dugong dugon*)^14^. An exception to this trend occurs in elephant seals (*Mirounga leonina*) which, in contrast to the aforementioned species, exhibit pronounced sexual size dimorphism hypothesized to drive observed differences in gut microbiomes between males and females^17^. Future studies of gut microbial communities in cetacean species that display drastic sexual size dimorphism (e.g. the true sperm whale, *Physeter macrocephalus*) will yield further insight into the impacts of sex-specific variations in body size and foraging behavior on gut microbiome structure. Importantly, no effect of carcass condition was recovered herein. These results are consistent with recent studies of postmortem mammal microbiomes (e.g. murine models)^44^ showing that endogenous gut bacteria continue to dominate gastrointestinal communities until advanced stages of decomposition, specifically intestinal rupture and the exposure of the abdominal cavity to oxygen. The postmortem stability of gut-associated communities indicates that stranded whales in early stages of decomposition can be used to provide accurate representations of cetacean gut microbiomes in wild ranging animals.

The taxonomic composition of kogiid gut microbiomes also offers insight into the putative functionality of gut-associated bacteria, as several core symbiont taxa were classified into bacterial lineages with known physiological capabilities. Facultative and obligate anaerobes with fermentative metabolism were common, including members of the families Ruminococcaceae (*Faecalibacterium*, *Oscillospira*), Enterobacteriaceae (*Citrobacter*) and Lachnospiraceae (*Buytrivibrio*). Microbial fermentation transforms undigested dietary components into end-products readily metabolized by mammal hosts, namely short-chain fatty acids (SCFA). Similar to ruminant mammals, cetacean gastrointestinal tracts include a non-glandular forestomach that likely functions as a fermentation vessel, based on cultivation studies of forestomach bacteria^45^, SCFA profiling of this chamber^46^ and metagenomic characterization of carbon metabolism in whales^25^. The taxonomic data herein, combined with past evidence of fermentation in sperm whales (based on bile acid composition)^47^, indicates that fermentative metabolism occurs in the gastrointestinal tract of *K*. *breviceps* and *K*. *sima* and potentially contributes to energy acquisition during prey digestion. Other core members of kogiid gut microbiomes were affiliated with sulfate reducing bacteria, including *Desulfovibrio* (Desulfovibrionaceae) and *Desulfomonile* (Syntrophaceae), which may persist in the anaerobic habitat of whale intestines by deriving energy from the respiration of sulfate present in small volumes of seawater ingested with prey. Finally, several putative pathogens were detected in *K*. *breviceps* and *K*. *sima*, including *Campylobacter fetus*, *Clostridium perfringens* and *Mycobacterium arupense*. The clinical relevance of these bacteria is unclear, although their role as opportunistic pathogens in other hosts^48^, taxonomic distinction from pathogens reported for toothed whale species^49^ and prevalence in diverse cetacean hosts^50^ suggest low virulence and potentially a greater threat to immunocompromised individuals.

In summary, this study characterized the structure and taxonomic composition of gut microbiomes of kogiid whales, a critical first step in understanding the role of cetaceans as microbial habitats and the contributions of symbiont communities to host metabolism and health. The selective pressures that contribute to the establishment and maintenance of host-specific microbiomes in kogiid whales may include unique microhabitats within each host, functional contributions of specific microbial guilds to host fitness, or a combination of both phenomena. Future studies targeting how these diverse, host-specific microbiomes form (e.g. comparative analyses across cetacean life stages) and their physiological characteristics (e.g. metagenomic and culture-based investigations) will provide further insight into microbiome development and functionality. Ultimately, a holistic understanding of host-microbe interactions in kogiid whales may yield insights into the impacts of gut dysbiosis on health and aid in health assessments and rehabilitation efforts for these species, which currently exhibit very low success rates due to mortality from gastric and intestinal stasis^35^.

## Methods

### Ethics Statement

All research activities were carried out under a NOAA Stranding Agreement to UNCW and research protocols were approved by UNCW’s Institutional Animal Care and Use Committee (protocols A0809-019, A1112-013 and A1415-015). There is considerable uncertainty surrounding the status of *Kogia breviceps* and *K*. *sima*, with both species categorized as “Data Deficient” on the IUCN Red List of Threatened Species (http://www.iucnredlist.org). This study relied solely upon postmortem sampling of stranded kogiid whales from North Carolina, responded to under authorization of the US Marine Mammal Protection Act. Animals were either found dead (*n* = 4), died during initial response (*n* = 4) or underwent humane euthanasia (n = 5) for reasons unrelated to this study following consultation with the National Marine Fisheries Service and under the supervision of a licensed veterinarian in accordance with the American Veterinary Medical Association Guidelines for the Euthanasia of Animals (2013 Edition).

### Sample Collection, DNA Extraction and Illumina Sequencing

**Figure 4:**
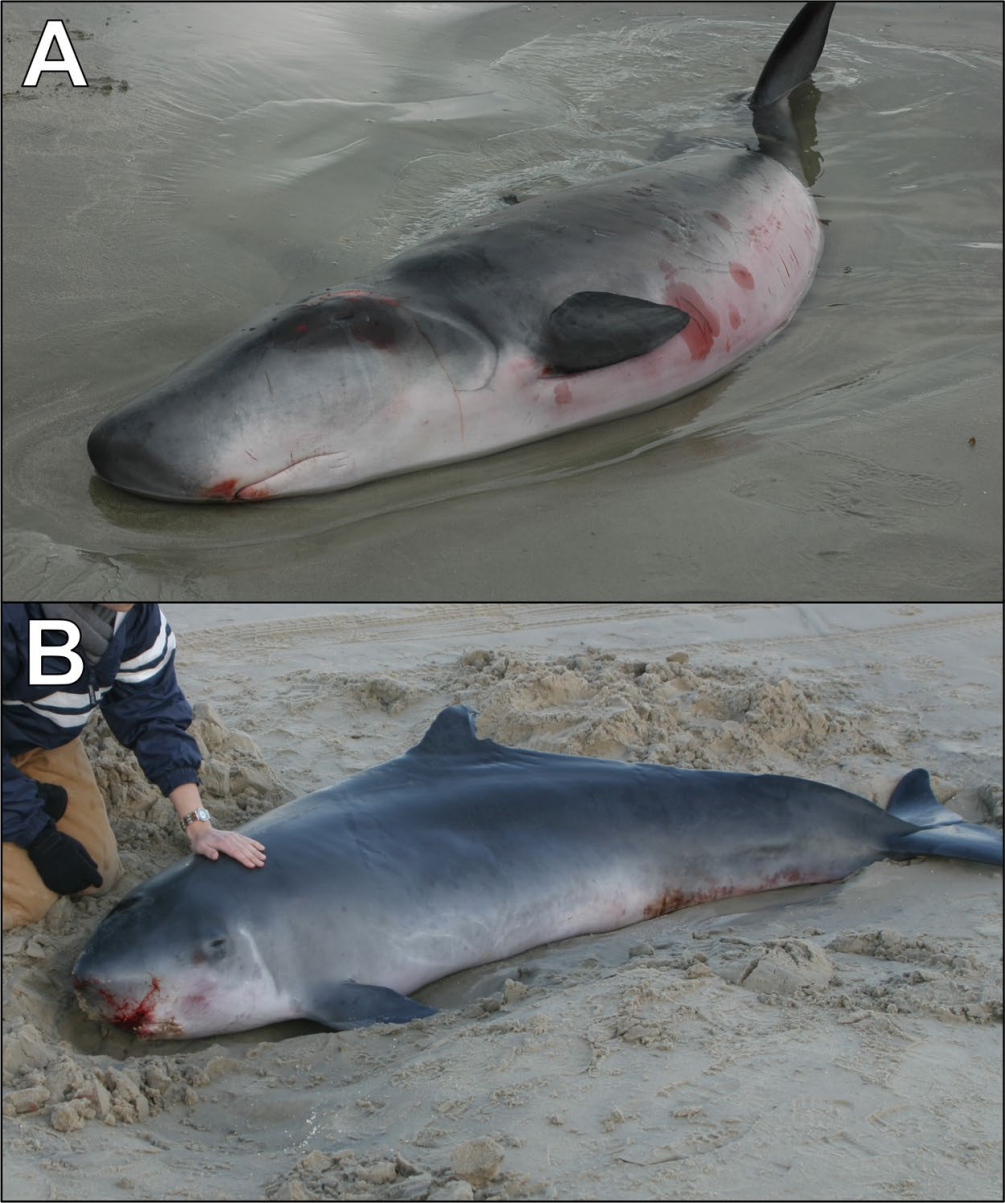
Stranded individuals of *Kogia breviceps* (**A**) and *K*. *sima* (**B**). Photo credits: UNCW Marine Mammal Stranding Program (**A**) and the Virginia Aquarium (**B**).

Colonic fecal samples were collected during necropsy of stranded individuals of *K*. *breviceps* (*n* = 9) and *K*. *sima* (*n* = 4) from the mid-Atlantic United States (North Carolina, Table 3, Fig. 4) between 2009 and 2014 and stored at −80 °C. All sampled individuals were adults (length range = 220–328 cm, weight range = 184–515 kg) stranded in fresh to moderate carcass condition (Carcass Classification Codes 1–3)^51^ and exhibited no signs of direct, human-induced mortality (e.g. boat collisions, fishery interactions). Sampled individuals included both sexes (Table 3).

**Table 3:** Stranded individuals of *K*. *breviceps* and *K*. *sima* examined in this study and associated metadata for each sample (n.a. = data not available).

Lab ID	Field ID	Species	Sex	Pregnant	Length (cm)	Weight (kg)	Strand Date	Latitude	Longitude	Condition^*^
K1	KLC-113	*K*. *breviceps*	F	Yes	286	n.a.	16-Sep-2011	36.04357 N	−075.67401 W	1, 2
K2	KLC-135	*K*. *breviceps*	F	No	252.5	183.6	5-Oct-2012	35.67985 N	−075.48023 W	1, 2
K3	NCARI-012	*K*. *breviceps*	F	Yes	296	n.a.	14-Oct-2011	36.41728 N	−075.83416 W	1, 2
K4	KLC-211	*K*. *breviceps*	F	No	295	314.8	16-Sep-2014	35.87716 N	−075.57759 W	1, 2
K5	KLC-106	*K*. *breviceps*	M	No	261	257.6	4-May-2011	36.04387 N	−075.67439 W	1, 2
K6	SWT-009	*K*. *breviceps*	M	No	328.5	515	9-Dec-2012	33.87536 N	−077.95721 W	1, 3
K7	WAM-644	*K*. *breviceps*	M	No	307	392	16-Aug-2008	33.90623 N	−078.34224 W	1, 2
K8	MDB-056	*K*. *breviceps*	M	No	263.5	n.a.	15-Dec-2009	35.71547 N	−075.49213 W	2, 3
K9	KLC-212	*K*. *breviceps*	M	No	293.5	418	1-Oct-2014	36.06975 N	−075.69198 W	1, 2
K10	CAHA-002	*K*. *sima*	F	No	233.5	n.a.	24-Aug-2010	35.35057 N	−075.49969 W	2, 3
K11	CAHA-065	*K*. *sima*	M	No	226	187.3	6-Jul-2011	35.77310 N	−075.52691 W	1, 2
K12	CAHA-003	*K*. *sima*	M	No	236.5	n.a.	24-Aug-2010	35.44597 N	−075.48259 W	2, 3
K13	CAHA-004	*K*. *sima*	M	No	220	n.a.	25-Aug-2010	35.45683 N	−075.48248 W	2, 3

^*^Carcass condition at stranding (left) and at examination (right): 1 = Alive, 2 = Fresh Dead, 3 = Moderate Decomposition.

DNA extracts were prepared from 200 mg of fecal material using the Powersoil DNA Extraction kit (MoBio), following Earth Microbiome Project standard protocols (http://press.igsb.anl.gov/earthmicrobiome/protocols-and-standards/dna-extraction-protocol/). DNA extracts were sent to Molecular Research LP (Shallowater, TX) for amplification, library construction and multiplexed sequencing of partial (V4) 16S rRNA gene sequences on an Illumina MiSeq platform. DNA extracts were used as templates for PCR amplifications using the HotStarTaq Plus Master Mix kit (Qiagen) and the universal bacterial/archaeal forward primer 515 f and reverse primer 806r^52^, with a multiplex identifier barcode on the forward primer. Thermocycler conditions consisted of an initial denaturation step at 94 °C for 3 min; 28 cycles of 94 °C for 30 s, 53 °C for 40 s, and 72 °C for 1 min; and a final elongation step at 72 °C for 5 min. Samples were pooled in equimolar concentrations, purified using Agencourt Ampure XP beads (Beckman Coulter) and sequencing on an Illumina MiSeq following the manufacturer’s guidelines. For comparative analyses, previously characterized cetacean gut microbiome datasets^25^ were downloaded for bottlenose dolphins (*Tursiops truncatus*), beluga whales (*Delphinapterus leucas*), humpback whales (*Megaptera novaeangliae*), right whales (*Eubalaena glacialis*) and sei whales (*Balaenoptera borealis*, Table S5). These datasets were constructed using the same extraction kit, primer pair and sequencing platform as the data generated herein.

### DNA Sequence Processing

Combined datasets from this study and Sanders *et al*.^25^ were processed simultaneously in mothur^53^ using a modified version (full details and mothur code in Table S6) of a previously described pipeline^54^. Briefly, sequence reads were quality-filtered, aligned to the SILVA database (release 119, non-redundant, mothur-formatted) and trimmed to the V4 region, screened for sequencing anomalies (e.g. chimeras) and errors, then assigned to taxonomic groups using a naïve Bayesian classifier^55^ and Greengenes taxonomy (May 2013 release, mothur-formatted). Following the removal of non-target amplicons (chloroplasts, mitochondria and eukaryotic reads), the remaining high quality sequences were clustered into operational taxonomic units (OTUs) at 97% sequence identity (average neighbor algorithm) and taxonomy assigned to each OTU by majority consensus^56^. Singleton OTUs (occurring once in the global dataset) were removed and datasets were subsampled to lowest read count (*n* = 24, 389) to avoid artifacts of varied sampling depth on diversity calculations. Raw sequence data were deposited as FASTQ files in the Sequence Read Archive of the National Center for Biotechnology Information (SRA NCBI) under the accession no. SRP097888.

### Statistical Analyses

To compare community diversity among cetacean hosts, alpha-diversity indices were calculated for OTU richness (observed richness, *S*), diversity (inverse Simpson, *1/D*), evenness (Simpson, *E*_*1/D*_) and dominance (Berger-Parker, *d*), as implemented in mothur. Analyses of variance (ANOVAs) were used to compare diversity index means across cetacean host species, followed by Tukey’s honest significant difference (HSD) tests for multiple pairwise post hoc comparisons.

To compare community structure among cetacean hosts, beta-diversity indices were calculated based on an OTU-dependent metric (Bray-Curtis similarity) and an OTU-independent (i.e. phylogenetic) metric (UniFrac distance)^57^. Two iterations of each beta-diversity metric were performed to differentiate between the effects of taxon abundance (OTU relative abundance Bray-Curtis, weighted UniFrac) and taxon membership (OTU presence-absence Bray-Curtis, unweighted UniFrac) on the structure of cetacean microbiomes. Bray-Curtis similarity values were calculated from OTU tables in PRIMER (version 6.1.11) and visualized in cluster dendrograms. UniFrac distances were calculated in mothur based on an approximate maximum-likelihood phylogeny constructed in FastTree 2.1.5^58^ with unique sequence reads (*n* = 52, 867). Permutational multivariate ANOVAs (PERMANOVA+, version 1.0.1) were used to test for differences in microbiome structure among host species, with significance determined by Monte Carlo asymptotic *P*-values corrected for multiple pairwise comparisons (Benjamini-Yekutieli false-discovery rate control^59^ and an experiment-wise error rate of α = 0.05), and to estimate components of variation ascribed to the factor ‘host species’ and to residual variation. Permutation multivariate analyses of dispersion (PERMDISP) were conducted to test for homogeneity of multivariate dispersions (i.e. deviations from centroids) among host species, with significance determined by permutational *P*-values similarly corrected for multiple pairwise comparisons.

Additional analyses of kogiid microbiome data were conducted to identify individual symbiont taxa contributing to community-level differences in microbiome structure between *K*. *breviceps* and *K*. *sima*. At the phylum level, significant differences in the relative abundance of bacterial phyla between kogiid hosts were determined using Student’s *t* tests. At the OTU level, a one-way similarity percentage species contributions (SIMPER) analysis was conducted to investigate the contribution of each symbiont OTU to the observed community dissimilarity between kogiid hosts. In addition, symbiont OTUs that were differentially abundant between kogiid hosts were identified using Metastats (non-parametric *t* tests)^60^ and LefSe (non-parametric Kruskal-Wallis sum-rank tests)^61^. To test for microbiome differentiation based on sex and carcass condition, separate two-way analyses of similarity (ANOSIM) were conducted for the factors ‘sex’ and ‘carcass condition’, each crossed with the factor ‘host species’ to control for differences between kogiid species. To exclude potentially transient members of kogiid symbiont communities, core microbiomes were identified for each kogiid host and strictly defined as symbiont OTUs detected in all samples within a host species.

## Electronic supplementary material

Supplementary Material

Supplementary Material
