# Supplementary material for: High diversity and unique composition of gut microbiomes in pygmy (Kogia breviceps) and dwarf (K. sima) sperm whales
Source: Sci Rep. 2017 Aug 3;7:7205. doi: 10.1038/s41598-017-07425-z (PMC5543158; doi:10.1038/s41598-017-07425-z)
Supplement: Supplementary file 1 — Supplementary Material [file 41598_2017_7425_MOESM1_ESM.pdf]

SUPPLEMENTARY MATERIAL FOR:

High diversity and unique composition of gut microbiomes in pygmy (*Kogia breviceps*) and dwarf (*K. sima*) sperm whales

Patrick M. Erwin*, Ryan G. Rhodes, Kevin B. Kiser, Tiffany F. Keenan-Bateman, William A. McLellan, D. Ann Pabst

Department of Biology and Marine Biology, Center for Marine Science, University of North Carolina Wilmington, Wilmington, NC 28409, USA

*Corresponding author: erwinp@uncw.edu, tel. 910-962-2326, fax 910-962-2410

Table S1. Relative abundance of bacterial and archaeal phyla in the gut microbiomes of *Kogia breviceps* and *K. sima*. Phyla exhibiting significant differences ($P < 0.05$) in relative abundance between hosts are indicated with an asterisk (*).

**Phylum**	***K. breviceps***	***K. sima***	***P***
Firmicutes	44.46 ±6.53	56.64 ±16.96	0.056
*Bacteroidetes	30.69 ±7.91	12.98 ±9.73	0.003
*Actinobacteria	6.08 ±3.81	14.69 ±6.03	0.007
Proteobacteria	5.00 ±3.51	6.71 ±7.65	0.499
Synergistetes	4.12 ±1.31	2.19 ±2.23	0.125
*Verrucomicrobia	2.69 ±1.62	0.22 ±0.32	0.019
Tenericutes	0.52 ±0.65	0.60 ±1.14	0.857
*Lentisphaerae	0.36 ±0.30	0.03 ±0.02	0.037
Euryarchaeota	0.07 ±0.27	0.01 ±0.01	0.309
Fusobacteria	0.05 ±0.08	0.01 ±0.01	0.434
Spirochaetes	0.03 ±0.02	0.01 ±0.01	0.118
*Cyanobacteria	0.003 ±0.003	0.041 ±0.039	0.011

Table S2. Pairwise statistical comparisons of multivariate dispersion (PERMDISP) across cetacean hosts, based on OTU-dependent (Bray Curtis) and OTU-independent (UniFrac) metrics of relative abundance (Rel. Abund., Weighted) and presence-absence (Presence-Abs., Unweighted) data. Asterisks (*) indicate significant differences following B-Y corrections. NT = no test.

	**Bray-Curtis Similarity**				**UniFrac Distance**			
	Rel. Abund.		Presence-Abs.		Weighted		Unweighted	
**Pairwise Comparison**	***t***	***P***	***t***	***P***	***t***	***P***	***t***	***P***
K. breviceps - K. sima	4.834	0.005*	2.364	0.056	3.329	0.004*	2.475	0.952
K. breviceps - D. leucas	0.752	0.864	35.913	0.013	0.823	0.764	0.227	1.000
K. breviceps - E. glacialis	0.933	0.444	5.642	0.001*	2.417	0.079	0.505	0.831
K. breviceps - M. novaeangliae	2.664	0.030	14.575	0.003*	3.757	0.013*	1.052	1.000
K. breviceps - T. truncatus	10.861	0.022	73.867	0.016	5.241	0.032	3.382	1.000
K. sima - D. leucas	2.853	0.254	46.561	0.075	1.782	0.408	1.974	0.659
K. sima - M. novaeangliae	0.955	0.551	9.843	0.033	0.920	0.450	2.215	0.134
K. sima - E. glacialis	3.192	0.017	2.993	0.012*	0.811	0.514	2.805	0.355
K. sima - T. truncatus	5.701	0.079	98.361	0.068	2.259	0.311	6.127	0.070
E. glacialis - D. leucas	0.021	1.000	9.172	0.020	0.808	0.750	0.565	1.000
E. glacialis - M. novaeangliae	1.582	0.288	3.951	0.008*	1.613	0.251	0.683	1.000
E. glacialis - T. truncatus	7.767	0.025	21.705	0.034	2.485	0.125	2.990	1.000
M. novaeangliae - D. leucas	1.233	0.789	6.804	0.095	1.773	0.227	0.737	0.685
M. novaeangliae - T. truncatus	4.932	0.192	21.904	0.095	0.589	0.694	1.175	0.698
T. truncatus - D. leucas	NT		NT		NT		NT	

Table S3. Statistical comparisons of microbial community similarity (ANOSIM) across sex and carcass condition of kogiid hosts, based on OTU-dependent (Bray Curtis) and OTU-independent (UNIFRAC) metrics of relative abundance (Rel. Abund., Weighted) and presence-absence (Presence-Abs., Unweighted) data. Two-way ANOSIMs were conducted to control for host species (source) effects (sex and species, carcass condition and species)

	**Bray-Curtis Similarity**				**UNIFRAC Distance**			
	Rel. Abund.		Presence-Abs.		Weighted		Unweighted	
**Factor**	***R***	***P***	***R***	***P***	***R***	***P***	***R***	***P***
Sex	-0.129	0.851	0.096	0.270	0.128	0.187	0.027	0.381
Carcass condition	0.025	0.389	0.103	0.368	-0.216	0.729	-0.285	0.868

Table S4. Core OTUs in the gut microbiomes of *K. breviceps* and *K. sima*. Values represent subsampled sequence counts by specimen. Differentially abundant OTUs were determined using Metastats and LEfSe ($P < 0.05$ for both tests).

Table provided as a separate data file.

Table S5. Sample and accession numbers of previously characterized cetacean gut microbiome data used in this study.

Sample	Metagenome	Species	Common Name	Group
JS16	BelugaWhale.JS16.16S.Ilm.V4	*Delphinapterus leucas*	Beluga Whale	Toothed
JS17	BelugaWhale.JS17.16S.Ilm.V4	*Delphinapterus leucas*	Beluga Whale	Toothed
JS13	BottlenoseDolphin.JS13.16S.Ilm.V4	*Tursiops truncatus*	Bottlenose Dolphin	Toothed
JS14	BottlenoseDolphin.JS14.16S.Ilm.V4	*Tursiops truncatus*	Bottlenose Dolphin	Toothed
JS10	HumpbackWhale.JS10.16S.Ilm.V4	*Megaptera novaeangliae*	Humpback Whale	Baleen
JS11	HumpbackWhale.JS11.16S.Ilm.V4	*Megaptera novaeangliae*	Humpback Whale	Baleen
JS9	HumpbackWhale.JS9.16S.Ilm.V4	*Megaptera novaeangliae*	Humpback Whale	Baleen
F11	RightWhale.F11.16S.Ilm.V4	*Eubalaena glacialis*	Right Whale	Baleen
F12	RightWhale.F12.16S.Ilm.V4	*Eubalaena glacialis*	Right Whale	Baleen
F16	RightWhale.F16.16S.Ilm.V4	*Eubalaena glacialis*	Right Whale	Baleen
F2	RightWhale.F2.16S.Ilm.V4	*Eubalaena glacialis*	Right Whale	Baleen
F5	RightWhale.F5.16S.Ilm.V4	*Eubalaena glacialis*	Right Whale	Baleen
F8	RightWhale.F8.16S.Ilm.V4	*Eubalaena glacialis*	Right Whale	Baleen
F9	RightWhale.F9.16S.Ilm.V4	*Eubalaena glacialis*	Right Whale	Baleen
JS1	SeiWhale.JS1.16S.Ilm.V4	*Balaenoptera borealis*	Sei Whale	Baleen

Table S6. Bioinformatics pipeline for raw sequence data processing in mothur (v.1.38.1), showing commands, input file types and settings for each step. Asterisks (*) indicate initial processing steps to integrate previous data from Sanders et al.[25]

Command	Input Files	Settings
*fastq.info	fastq	none
*reverse.seqs	fasta	none
*merge.files	fasta	none
*screen.seqs	fasta, group	maxambig=0, maxlength=300, minlength=200, maxhomop=8
trim.seqs	fasta, group	maxambig=0, maxlength=300, minlength=200, maxhomop=8, bdiffs=0, pdiffs=2
*merge.files	fasta	input=, output=
*merge.files	group	input=, output=
unique.seqs	fasta	none
align.seqs	fasta, reference	none
screen.seqs	fasta, group, name	start=13862, end=23444
filter.seqs	fasta	vertical=T, trump=.
pre.culster	fasta, group, name	diffs=2
chimera.uchime	fasta, group, name	dereplicate=t, reference=self
remove.seqs	fasta, group, name	accnos=
classify.seqs	fasta, group, name	reference= , taxonomy = , cutoff=60
remove.lineage	fasta, group, name, taxonomy	taxon=Chloroplast-mitochondria-unknown-eukaryota
filter.seqs	fasta	vertical=T, trump=.
cluster.split	fasta, name, taxonomy	splitmethod=classify, taxlevel=4, cutoff=0.10
remove.rare	list, group	nseqs=1, label=0.03
classify.otu	list, name, taxonomy	label=0.03
get.oturep	fasta, group, name, list	method=abundance, label=0.03
make.shared	list, group	label=0.03
sub.sample	list, group	size=24389, persample=t
list.otulabels	list	none
get.otulabels	accnoss, constaxonomy	none
make.shared	list, group	none

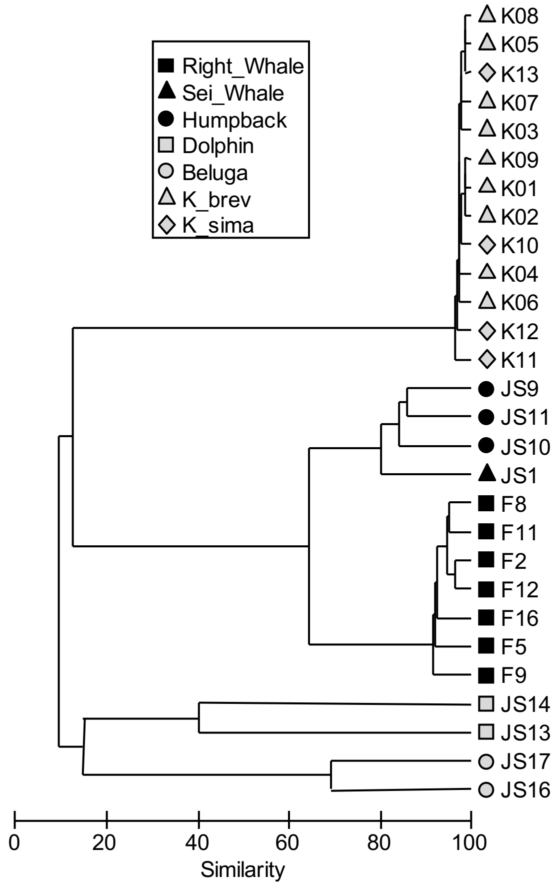

Figure S1. Similarity of gut microbiomes in *Kogia breviceps* and *K. sima* compared with previous data from other toothed and baleen whales based on presence-absence OTU data. Gray symbols indicate toothed whale species and black symbols denote baleen whale species.
